# ﻿*Vacciniumdehongense* (Ericaceae), a new species of *Vaccinium* sect. *Epigynium* from western Yunnan, China

**DOI:** 10.3897/phytokeys.242.121623

**Published:** 2024-05-13

**Authors:** Yi-Hua Tong, Xing-Er Ye, Jing-Bo Ni

**Affiliations:** 1 State Key Laboratory of Plant Diversity and Specialty Crops/Key Laboratory of Plant Resources Conservation and Sustainable Utilization, South China Botanical Garden, Chinese Academy of Sciences, Guangzhou, 510650, China South China Botanical Garden, Chinese Academy of Sciences Guangzhou China; 2 Key Laboratory of National Forestry and Grassland Administration on Plant Conser-vation and Utilization in Southern China, Guangzhou 510650, China Key Laboratory of National Forestry and Grassland Administration on Plant Conser-vation and Utilization in Southern China Guangzhou China; 3 South China National Botanical Garden, Chinese Academy of Sciences, Guangzhou, 510650, China South China National Botanical Garden, Chinese Academy of Sciences Guangzhou China; 4 School of Chinese Medicinal Resource, Guangdong Pharmaceutical University, Yunfu, 527325, China Guangdong Pharmaceutical University Yunfu China

**Keywords:** Morphology, taxonomy, Vaccinieae, Yingjiang County

## Abstract

*Vacciniumdehongense* (Ericaceae), a new species from Yingjiang County of Yunnan Province, China is described and illustrated. This new species belongs to Vacciniumsect.Epigynium and is most similar to *V.vacciniaceum*, but differs from the latter in the subsessile leaves, the inflorescence usually developing at leafless nodes, the shorter pedicels and the filaments being ca. 1/3 length of the stamens. Since the type locality of this new species is very near the border between China and Myanmar, it is probably also distributed in the adjacent area of Myanmar. As no population assessment of this species in its whole distribution area is made, it is best to assign a conservation status of ‘Data Deficient’ (DD) for this species.

## ﻿Introduction

China, with more than 41,000 species of higher plants, is one of the countries owning the highest plant diversity in the world (Xie et al. 2021). Even now, more than 280 new species were described from China each year from 2020 to 2022 (Du et al. 2023). Collaboration between experts and investigators is one of the factors that accelerates the findings of new species. As the taxonomic study of *Vaccinium* L. (Ericaceae) in China is continuously being undertaken, the number of specie of this genus from this country now reaches 103 (Guo et al. 2023; Qin et al. 2023; Tong et al. 2023).

During several field trips to Yingjiang County of Yunnan Province, China, we encountered an unknown *Vaccinium* species. Its pseudo-verticillate leaves indicate that it should belong to V.sect.Epigynium (Klotzsch) Hook. f. Only one formerly known species of this section, recorded from western Yunnan, is *V.scopulorum* W. W. Sm., which has an alternative phyllotaxis rather than a pseudo-verticillate one. After a careful comparison with similar species of the same section from China and adjacent countries (Rae 1991; Kress et al. 2003; Fang and Stevens 2005; Panda and Sanjappa 2014; Holt and Maden 2022), it was confirmed that this species is new to science, which is described and illustrated below.

## ﻿Materials and methods

Specimens were collected from Yingjiang County in two field trips in June 2015 and March 2023, respectively. Descriptions were based on dried collections, except the information of flower colour. Measurements were performed with a ruler and small plant parts were observed and measured under a stereomicroscope (Mshot-MZ101). General terminology follows Beentje (2016).

## ﻿Taxonomic treatment

### ﻿ Vaccinium dehongense

Taxon classificationPlantaeEricalesEricaceae

Y.H.Tong sp. nov.

B73D390E-AC6C-5790-9755-C3F660CCDA70

urn:lsid:ipni.org:names:77341761-1

Fig. 1

#### Type.

China. Yunnan Province: Dehong Dai and Jingpo Autonomous Prefecture, Yingjiang County, Xima Township, Huoshigou Village, epiphytic on trees in evergreen broad-leaved forest, 24°47'5.4"N, 97°43'35.8"E, 1740 m a.s.l., 12 March 2023 (fl.), *Yi-Hua Tong et al. TYH-2651* (holotype: IBSC; isotypes: KUN, PE).

**Figure 1.**
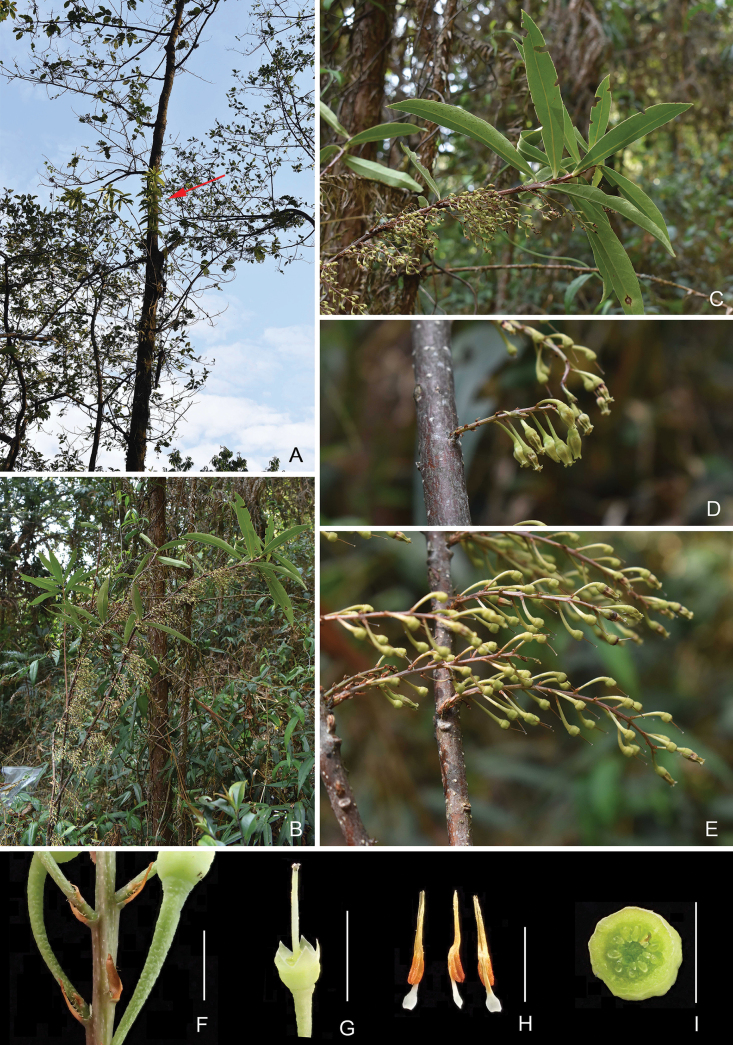
*Vacciniumdehongense***A** habitat, the red arrow indicating this species **B** habit **C** flowering branchlets **D** inflorescences **E** infructescences with immature fruits **F** part of an inflorescence, showing bracts and bracteoles **G** flower with corolla removed **H** stamens, adaxial, lateral and abaxial view **I** ovary cross-section, showing pseudo-10-locular ovary. Scale bars: 5 mm (**F–G**); 3 mm (**H–I**). Photographs by Yi-Hua Tong.

#### Diagnosis.

This new species is morphologically similar to *V.vacciniaceum*, especially its subspecies, V.vacciniaceumsubsp.glabritubum P. F. Stevens (with a glabrous internal corolla surface), in having pseudo-verticillate leaves with a serrate margin and a rounded leaf base, glandular-setulose twigs with scattered lenticels, elongate racemose inflorescences with many flowers, narrowly triangular bracts and bracteoles and a glabrous internal corolla surface, but can be distinguished by subsessile (vs. with 1–4 mm long petioles) leaves, the inflorescences usually developing at leafless nodes (vs. axils of leaves), shorter pedicels (6–7.5 mm vs. 7–13 mm) and filaments being ca. 1/3 length of the stamens (vs. ca. 1/2).

#### Description.

Evergreen shrubs, 0.5–3 m tall, epiphytic on trees, with inflated root tubers. Young twigs yellowish-brown, angled, densely glandular-setulose, glabrescent; old branches purple brown, with sparse white lenticels. Bud scales subulate, margin glandular-ciliate. Leaves often 4–8-pseudoverticillate, subsessile, borne on a protuberance; blades firmly papery, oblong-lanceolate, 5.5–15 × 0.8–3 cm, apex acute to acuminate, base cuneate or slightly obtuse, margin plane, serrate, each serra tipped with a gland, glabrous on both surfaces, mid-vein slightly raised adaxially, strongly raised abaxially, lateral veins 11–14 on each side, together with fine veins raised on both surfaces. Inflorescences racemose usually at leafless nodes, rarely axillary, 2–8 cm long, with 7–21 flowers, rachis glabrous, with persistent bud scales at base; bracts narrowly triangular, 1.5–2.5 × 0.2–0.5 mm, glabrous on both sides, margin glandular-ciliate; pedicel 6–7.5 mm long, glabrous, thickened upwards; bracteoles 2, caducous, usually borne near the base of the pedicel, occasionally at the lower part, shape and indumentum similar to bracts, but smaller, ca. 1 × 0.2 mm. Hypanthium 0.8–1 × 1.2–1.5 mm, glabrous, smooth when fresh, rugose when dry; calyx limb 5–lobed to near base, lobes greenish-yellow, sometimes tinged with purplish, triangular to ovate-triangular, 1–1.5 × 1.2–1.5 mm, glabrous, apex acuminate. Corolla greenish-yellow, urceolate, 5-angled, 5–6 × 2–2.5 mm, glabrous on both surfaces, apex shallowly lobed, lobes recurved, triangular, ca. 1–1.2 × 1 mm, abaxially glabrous, adaxially papillose; stamens 10, 4.5–5 mm long, filaments flat, slightly expanded at base, 1.5–1.6 mm long, glabrous; anthers 3–4 mm long, thecae 1–1.2 mm long, more or less echinate on edges, with 2 small appendages at base, tubules 2–2.8 mm long, opened by a long slit more than half of the tubules, dorsal spurs absent; style 4.5–5.5 mm long, stigma slightly expanded, capitate; ovary pseudo-10-locular, each locule with several ovules, disc glabrous. Fruit unknown.

#### Etymology.

The species epithet is derived from the type locality, Dehong Dai and Jingpo Autonomous Prefecture. Its Chinese name is given as 滇西越橘 (Pinyin: diān xī yuè jú).

#### Distribution and habitat.

This species is currently known only from the type locality, i.e. Yingjiang County, Dehong Dai and Jingpo Autonomous Prefecture, Yunnan Province. It grows on trees in mountainous evergreen broad-leaved mixed forests at elevations of 1400–1750 m a.s.l.

#### Conservation status.

Since the type locality of this new species is very near the border between China and Myanmar, it is probably also distributed in the adjacent area of Myanmar. As no population assessment of this species in its whole distribution area is made, it is best to assign a status of ‘Data Deficient’ (DD) for this species following the IUCN Red List Categories and Criteria (IUCN Standards and Petitions Committee 2022).

#### Phenology.

Flowering in March.

#### Discussion.

*Vacciniumdehongense* obviously belongs to V.sect.Epigynium due to its evergreen and pseudo-verticillate leaves with a serrate margin, urceolate corollas and stamens without spurs on the back of anthers (Sleumer 1941; Stevens 1969; Vander Kloet and Dickinson 2009). In the key to *Vaccinium* in the *Flora of China* (Fang and Stevens 2005), *V.dehongense* is keyed out to be close to *V.vacciniaceum*. The main differences between the two species are indicated in the diagnosis part. The most distinct character that distinguishes the two species is the position where inflorescences develop. A more detailed comparison between the two species is presented in Table 1. Besides, they have an allopatric distribution: *V.dehongense* is endemic to west Yunnan, China, while *V.vacciniaceum* is distributed in northwest Myanmar, northeast India, SE Xizang of China, Bhutan and Nepal. Panda and Sanjappa (2014) merged V.vacciniaceumsubsp.glabritubum to the nominate subspecies without giving any reason. However, just as Stevens (1985) pointed out, V.vacciniaceumsubsp.glabritubum has subsessile leaves with a round base and a glabrous corolla, while the nominate subspecies owns obviously petiolate leaves with a cuneate base and a hairy internal corolla surface. Besides, V.vacciniaceumsubsp.glabritubum is distributed in Nepal, Sikkim, Bhutan and SE Xizang of China and the nominate subspecies has a further southeast distribution, viz. Meghalaya, Nagaland, Manipur, Mizoram of India and Chin State of Myanmar. Considering the morphological differences and allopatric distribution pattern of the two subspecies, it is better to treat them as two distinct subspecies for now, until new evidence (such as molecular evidence) is obtained.

In the type locality, there is another species from the same section, i.e. *V.scopulorum* W. W. Sm., which is much more common than *V.dehongense* and has a wider distribution including west China, Myanmar and Bhutan. *Vacciniumscopulorum* also has setose branchlets, but its alternate phyllotaxis, smaller leaf blades (2.5–4.5 × 0.6–1.7 cm) and yellowish-green corollas with a dark-purple apex are very different from those of *V.dehongense*.

**Table 1.** A comparison of *Vacciniumdehongense* and *V.vacciniaceum*. The data of the latter species are taken from Wight (1850), Stevens (1985), Fang and Stevens (2005) and the examined specimens listed in the text.

Comparison items	*V.dehongense*	*V.vacciniaceum*
Twigs	Angled	Round
Petiole length (mm)	Subsessile	1–4
Inflorescence position	Usually at leafless nodes, rarely axillary	Axillary
Pedicel length (mm)	6–7.5	7–13
Corolla colour	Greenish-yellow	Greenish-white or pinkish-yellow
Ratio of filament length to stamen length	Ca. 1/3	Ca. 1/2
Filament indumentum	Glabrous	Pubescent or subglabrous
Distribution	West Yunnan of China	Bhutan, northeast India, south Xizang of China and north Myanmar

#### Additional specimens examined.

*Vacciniumdehongense* (paratypes): CHINA. Yunnan Province: Dehong Dai and Jingpo Autonomous Prefecture, Yingjiang County, Xima Xiang, 11 June 2015, *Yi-Hua Tong & Xing-Er Ye TYH-128* (IBSC), *TYH-129* (IBSC); ibid., No. 2 water power station of Mengnai River, 12 March 2023, *TYH-2659* (IBSC).

Vacciniumvacciniaceumsubsp.vacciniaceum: INDIA. Meghalaya State: Garrow [Garo] hills, 1813, *W. Roxburgh s.n.* (holotype BM000802681, image); Silhit Mountains [Khasi hills], *W. Gomez 6299* (K000780682, image); ibid., 4000 ft [1219 m a.s.l.], *J. D. Hooker & T. Thomson s.n.* (K000780683, image); ibid., *W. Griffith s.n.* (K000780684, image; M-0164586, image); without precise locality, *J. O. Viogt 252* (IBSC0457574).

Vacciniumvacciniaceumsubsp.glabritubum: BHUTAN. Chhukha State: 13 km SW of Gedu between Phuntsholing and Gedu, 1780 m a.s.l., *B. Bartholomew & D. E. Boufford 3940* (PE00197369). NEPAL. Arun Valley, Maghang Kola, E of Num, 9000 ft [2743 m a.s.l.], 30 April 1956, *Stainton 167* (holotype A00015998, image; isotype BM000802680, image). INDIA. Sikkim State: *J. D. Hooker s.n.* (E00438126, image).

## Supplementary Material

XML Treatment for Vaccinium dehongense

## ﻿Additional information

### Author ORCIDs

Yi-Hua Tong https://orcid.org/0000-0002-5034-005X

### Data availability

All of the data that support the findings of this study are available in the main text.
